# Successful pregnancy after complete resection of leiomyomatosis peritonealis disseminate without recurrence:a case report with next-generation sequencing analysis and literature review

**DOI:** 10.1186/s12957-020-01857-0

**Published:** 2020-05-02

**Authors:** Hualei Bu, Chengjuan Jin, Yan Fang, Yana Ma, Xiao Wang, Jingying Chen, Lijun Chen

**Affiliations:** 1 Department of Obstetrics and Gynecology, Qilu Hospital of Shandong University, 107 Wenhua Xi Road, Jinan, 250012 People’s Republic of China; 2 Department of Obstetrics and Gynecology, Shanghai General Hospital, School of Medicine, Shanghai Jiao Tong University, 650 XinSongjiang Road, Shanghai, 201620 People’s Republic of China; 3 Department of Pathology, Qilu Hospital of Shandong University, 107 Wenhua Xi Road, Jinan, 250012 People’s Republic of China

**Keywords:** LPD, NGS, Pregnancy, Leiomyosarcoma, The authors Hualei Bu and Chengjuan Jin contributed equally to this work.

## Abstract

**Background:**

Peritoneal leiomyomatosis disseminate (LPD) is a rare disease characterized by widespread dissemination of leiomyomas nodules throughout the peritoneal and omental surfaces. Reports of pregnancy with LPD are even rarer. Therefore, there is no clear consensus on the treatment of LPD on pregnancy, and the pathogenesis is still unclear.

**Case presentation:**

We reported a case of LPD patient who developed during pregnancy. The patient underwent a cesarean section at 32 weeks of gestation while removing all visible tumors, and no LPD lesions were seen in the subsequent cesarean section at full term. NGS of LPD lesions detected 4 mutations with focal high-level amplifications of CDK4 (cyclin-dependent kinases 4), NBN (Nibrin), DAXX (death domain associated protein), and MYC (myelocytomatosis oncogene). Immunohistochemistry staining analysis among benign leiomyoma, LPD, and leiomyosarcoma verified that LPD was an unusual intermediate between benign and malignant uterine smooth muscle tumors. Besides, LPD is a hormonal-dependent leiomyoma. After a detailed literature search, we summarized the detailed clinical features and follow-up information of patients with LPD during pregnancy.

**Conclusions:**

This is the first reported LPD case of successful term pregnancy without recurrence, following resection of all visible lesions in a prior pregnancy. LPD is an unusual intermediate between benign and malignant uterine smooth muscle tumors.

## Introduction

Uterine smooth muscle tumors include a variety of tumors, such as benign uterine leiomyoma, malignant leiomyosarcoma, and tumors with unusual growth patterns. Uterine leiomyoma is the most common tumor of the female reproductive system [1]. Benign leiomyoma variants mainly include atypical leiomyoma, plexiform leiomyoma, cellular leiomyoma, and smooth muscle tumor of uncertain malignant potentia l[2]. Leiomyosarcoma is a uterine malignancy with an aggressive clinical behavior and poor prognosis. Leiomyosarcoma distinguishes from uterine leiomyoma by the presence of coagulative tumor necrosis, severe cellular atypia, extreme cytogenetic instability, and elevated mitotic activity [3]. LPD as well as intravenous leiomyomatosis belongs to a class of tumors resembling uterine leiomyoma at both gross and microscopic levels but presenting in unusual locations with recurrent and malignant tendencies [4].

LPD is a rare benign intra-abdominal leiomyoma characterized by multifocal proliferation of smooth muscle-like cells that are histologically similar to uterine leiomyoma [5, 6]. Up to date, there have been no more than 200 cases published, of which approximately half been reported in child-bearing years and only few cases in postmenopausal women [7–9]. LPD lesions always involve the pelvic, the abdominal peritoneum, and the omentum. The patients generally present with no clinical symptoms; however, abdominal pain or abdominal distension do occasionally occur [10]. Clinical examination usually reveals numerous smooth muscle nodules in the pelvic, the abdominal peritoneum, and the omentum. Histopathology examination suggests benign uterine smooth muscle tumors, rare mitotic activity, and without nuclear atypia [11].

However, there is still no standardized guideline for the diagnosis and treatment of LPD. LPD during pregnancy is even rarer and has been reported only in limited cases, so there is no definite consensus about the adverse effects of LPD on pregnancy and the safety of re-pregnancy for women with a history of LPD. In this study, we reported a patient with LPD that occurred during pregnancy. All LPD lesions were removed in the cesarean section, and there was no relapse of LPD in the subsequent pregnancy. We then reviewed relevant literature and summarized the obstetric-related clinical information and follow-up information of LPD patients who occurred during pregnancy, hoping to provide a theoretical basis for the treatment of LPD.

## Case presentation

### Case

A 19-year-old woman with 32^+3^ weeks of gestation was referred to our hospital due to oligohydramnios. The patient had a history of myomectomy at age 15. At that time of ultrasound examination, there was a mass of 20.0 cm × 8.7 cm in size in the pelvic cavity. Postoperative pathological findings showed cellular uterine leiomyoma.

**Fig. 1:**
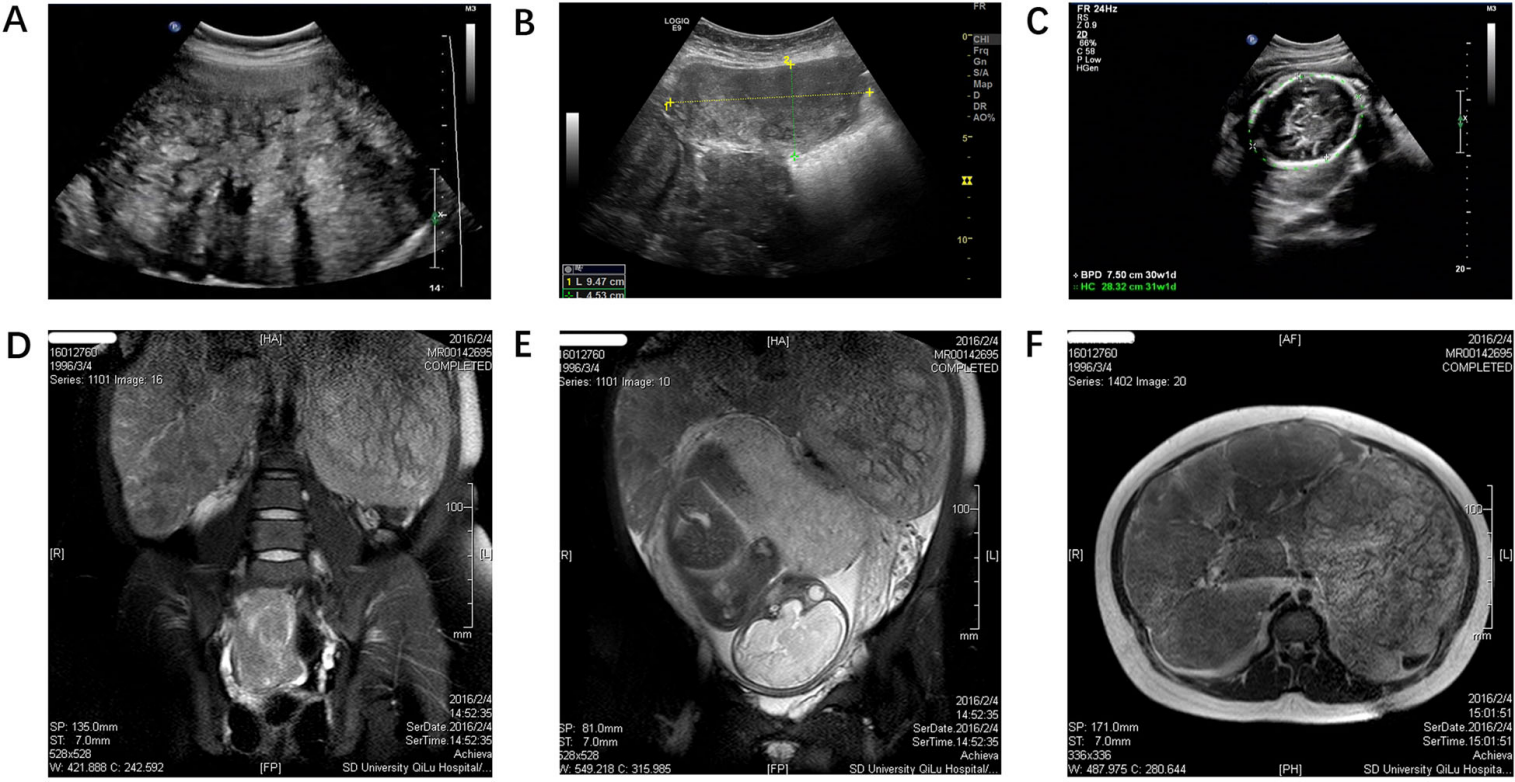
Ultrasound and MRI showed the presence of multiple masses in the pelvis. **a**, **b** Ultrasound showed huge mass in the pelvis. **c** Fetal head in ultrasound. **d** Multiple huge masses in the pelvis were shown in MRI. **e**, **f** The fetal was squeezed by huge masses in MRI

On admission, both the patient and the fetus were in good condition. Physical examination revealed a huge mass in the pelvic cavity. Abdominal and pelvic ultrasound confirmed the presence of multiple masses in the pelvic, sized 16.9 × 11.2 × 10.1 cm, 13.1 × 5.6 × 6.2 cm, and 19.2 × 17.5 × 12 cm, respectively, next to the gestation without signs of abortion. The masses were connected into large clumps. An abdominal MRI was done to show multiple nodules in the abdominal cavity (Fig. 1).

**Fig. 2:**
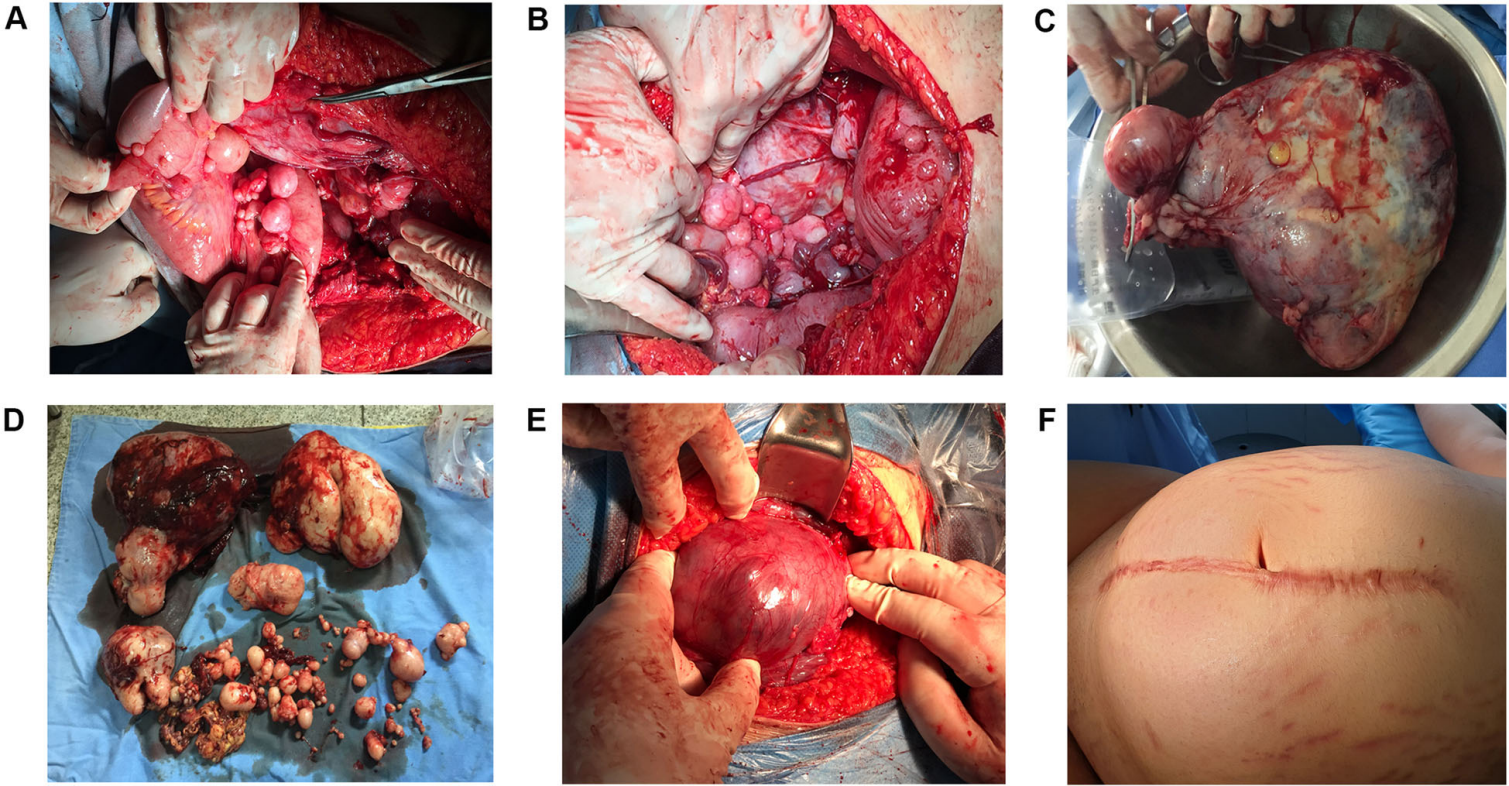
Gross features of LPD during laparotomy. **a**, **b** Concentrated myoma tubercle-like cysts on the surface of the uterine, the intestine, and mesentery. **c** The resected huge myoma. **d** All myomas removed in laparotomy, two large myomas, two moderate myomas, and multiple small myomas. **e** A single uterine fibroid was found in the posterior wall of the uterus in the second cesarean section. **f** Abdominal scar of the first cesarean section

In order to ascertain the diagnosis, an exploratory laparotomy was performed because of aggravated abdominal pain. After the delivery of the fetus by lower-segment cesarean section, the gynecological oncologist performed further operation. The patient was found to have multiple sporadic leiomyoma in the anterior wall of the uterus; an 8 × 6 cm leiomyoma in the posterior wall of the uterus; a 20 × 15 cm tumor mass in the left pelvis; and multiple tumor masses in the right pelvic sized 8 × 7 cm, 7 × 7 cm, and 7 × 5 cm separately up to 10 tumor masses sized 3 × 2 cm in the omentum and mesocolon transversum (Fig. 2a–d). All macroscopic tumor masses were dissected and removed via an extremely difficult surgery without hysterectomy and bilateral salpingo-oophorectomy because of the patient’s strong objection and the consideration of young age. Post-operative pathology determined the diagnosis of LPD with red degeneration. The patient recovered well after surgery and was discharged on the ninth day after the removal of the abdominal incision suture.

The patient underwent several ultrasound examinations after surgery, and no signs of disease recurrence were found without any continuous treatment. The patient was pregnant again 25 months after the surgery. At 7 weeks of gestation, ultrasound examination revealed a fibroid of about 3.7 cm × 3.7 cm in the posterior wall of the uterus, and ultrasound examination during pregnancy indicated that the fibroid was slowly enlarged without any discomfort symptoms. The patient underwent a cesarean section again at 39 weeks of gestation. No abnormal lesions were found in the pelvic and abdominal cavity during the operation, and only a uterine fibroid of about 7 cm × 6 cm was found in the posterior wall of the uterus (Fig. 2e). Postoperative pathology suggested uterine leiomyoma. The patient was reviewed at 6 months postoperatively and recovered well.

### NGS (next-generation sequencing)

We collected 15- of 4-μm tissue sections from formalin-fixed paraffin-embedded (FFPE) samples of LPD and normal tissue adjacent to the lesion for the genetic analyses. QIAamp DNA FFPE Tissue Kit (QIAGEN, Heidelberg, Germany) was used to extract genomic DNA according to the manufacturer’s instructions.

DNA was profiled using a commercial available capture-based targeted sequencing panel (Burning Rock Biotech, Ltd., Guangzhou, China), targeting 295 genes which were closely related to the mechanism of cancer and targeted therapy and spanning 1.5 MB of human genomic regions. DNA shearing, end-repair, and adaptor ligation were performed by the use of Covaris M220 (Covaris, Inc., MA, USA). Fragment sizes ranging from 200 to 400 bp were selected using Agencourt AMPure beads (Beckman Coulter, CA, USA) followed by hybridization with capture probes baits, hybrid selection with magnetic beads, and PCR amplification. Subsequently, Qubit® 3.0 and Agilent 2100 bioanalyzer (Agilent Technologies Inc., CA, USA) was performed to assess the quality and size of the fragments. Indexed samples were sequenced on Nextseq500 sequencer (Illumina, Inc., CA, USA) with pair-end reads.

**Fig. 3:**
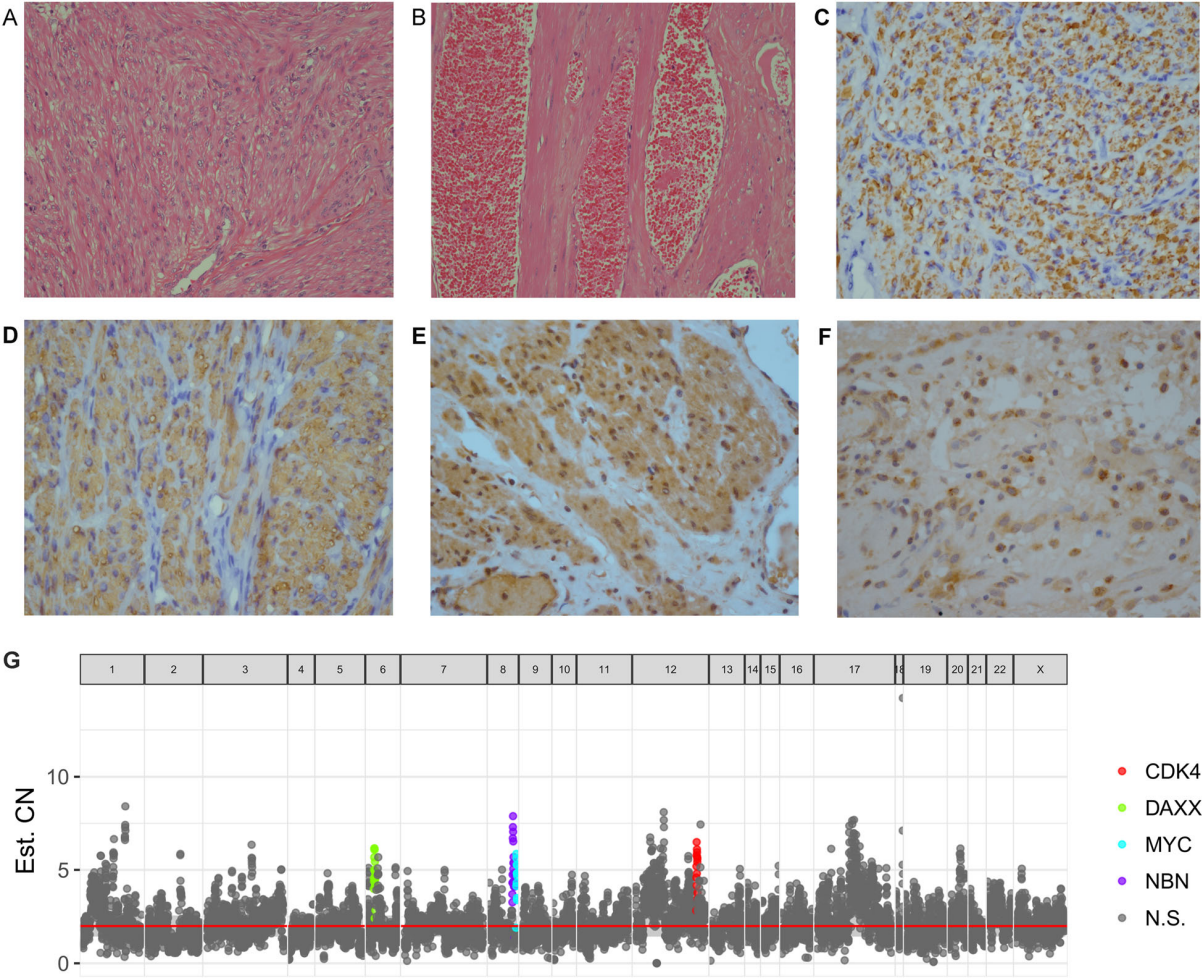
HE staining and immunohistochemistry analysis of LPD. **a**, **b** HE staining of this LPD, suggesting benign myoma with rich blood supply. **c** Immunohistochemistry staining of Desmin, × 40. **d** Immunohistochemistry staining of SMA, 40 ×. **e** Immunohistochemistry of estrogen receptor (ER), × 40. ER was strongly positive in LPD. **f** Immunohistochemistry of progesterone receptor (PR), × 40. PR was strongly positive in LPD. **g** Distribution plot of gene copy number in NGS of this LPD. CDK4, DAXX, MYC, and NBN were significantly amplified (red = CDK4, green = DAXX, blue = MYC, purple = NBN)

Based on the high throughput sequencing, the copy numbers (CNs) of this LPD patient compared with the normal population were demonstrated in Fig. 3g. There were four somatic cell line mutations detected in the lesions. The CNs of CDK4, NBN, DAXX, and MYC were all amplified for at least 4 times.

### Hematoxylin-eosin (HE) and immunohistochemistry staining

Hematoxylin-eosin (HE) staining slides of this LPD were shown in Fig. 3a. Rich blood supply was revealed in LPD in HE staining analysis (Fig. 3b).

Immunohistochemistry staining showed that the tumor was strongly positive for smooth muscle markers, SMA and Desmin (Fig. 3c, d), which suggested that LPD shared partial molecular cytogenetic characteristics with uterine leiomyoma. Immunohistochemistry of hormone receptors, estrogen receptor (ER), and progesterone receptor (PR) was positive (Fig. 3e, f).

**Fig. 4:**
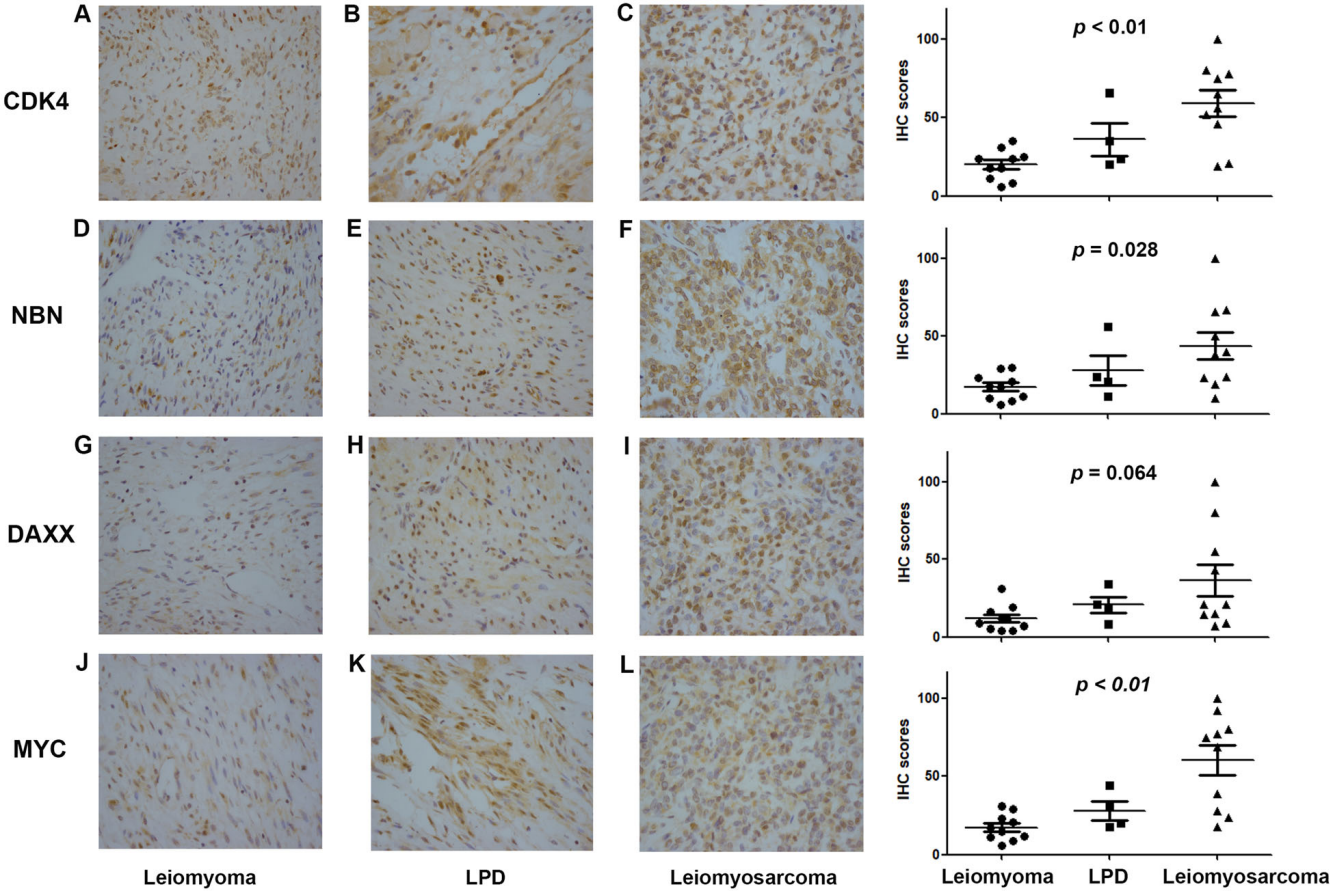
Immunohistochemistry staining analysis of CDK4, NBN, DAXX, and MYC in leiomyoma, LPD, and leiomyosarcoma, suggesting that LPD is an unusual intermediate between benign and malignant uterine smooth muscle tumors

The immunohistochemistry staining analysis of CDK4, MYC, NBN, and DAXX in uterine leiomyoma (10 cases), LPD (4 cases), and leiomyosarcoma (10 cases) was subsequently conducted. The uterine leiomyoma tissues were obtained from patients who underwent hysteromyomectomy and proved to have no malignant lesions. The clinical information of LPD and leiomyosarcoma patients was seen in Tables 1 and 2. We defined the scores of staining intensities as 0 (negative), 1 (weak), 2 (moderate), and 3 (strong) and then multiplied with the corresponding area to obtain the scores of immunohistochemistry. The highest score for each group was defined as 100, and the other scores were converted accordingly. The results revealed that the expression profiles of LPD were more similar to leiomyosarcoma. LPD showed CDK4, NBN, DAXX, MYC moderately, and strongly positive, and uterine leiomyosarcoma displayed strongly positive. However, the four markers in uterine leiomyoma were slightly positive or negative (Fig. 4). Therefore, we can infer the conclusion that LPD is an intermediate disease between benign uterine fibroids and malignant leiomyosarcoma.

**Table 1:** Clinical information of LPD patients for immunohistochemical analysis

No.	Age	Obstetric history	History of hysteromy-omectomy	Assisted reproductive technology	Operative methods	Menstrual status
1	32	G2P1	Yes	No	Lesions resection	Premenopausal
2	46	G3P1	Yes	No	Lesions resection and bilateral salpingo-oophorectomy	Premenopausal
3	40	G2P2	Yes	No	Lesions resection	Premenopausal
4	19	G1P0	Yes	No	Lesions resection	Premenopausal

**Table 2:** Clinical information of uterine leiomyosarcoma patients for immunohistochemical analysis

No.	Age	Obstetric history	FIGO	Size, maximum dimension (cm)	Adjuvant chemotherapy	Menstrual status
1	45	G3P1	IB	17	No	Premenopausal
2	26	G2P1	IIB	8	No	Premenopausal
3	37	G3P1	IB	10	No	Premenopausal
4	31	G1P1	IA	3	No	Premenopausal
5	44	G2P1	IIB	12	No	Premenopausal
6	44	G5P2	IB	8	No	Premenopausal
7	43	G3P1	IIIB	4.3	No	Premenopausal
8	46	G4P1	IB	9	No	Premenopausal
9	38	G1P1	IB	12.5	No	Premenopausal
10	48	G3P1	IB	10	No	Premenopausal

## Discussion and conclusions

In 1952, Willson and Peale described LPD for the first time [6]. LPD is characterized with multiple nodules in various sizes in the peritoneal cavity, such as the uterus, fallopian tubes, intestine, mesentery, omentum, and retroperitoneum [6]. The incidence of LPD was unkown due to its rarity. There have been no more than 200 cases reported in the literature up to date.

LPD was difficult to diagnose before surgery. Although it was a benign disease with an excellent prognosis, LPD could behave quasi-malignant behavior, such as recur tendency and spread widely in the pelvic and abdominal cavity. LPD should be differentiated from peritoneal metastasis of malignancies. Standard histopathological analysis as well as immunochemistry was in need to diagnose LPD accurately. Microscopically, the knots are composed of smooth muscle arranged like leiomyomas, and the cells usually show a lack of atypia and higher mitotic variety [9]. In this study, the patient was suspected to have malignant tumors in the pelvic and peritoneal cavity initially and was eventually diagnosed with LPD by histopathology. LPD must be distinguished from malignancies to avoid unnecessary aggressive treatment schedules.

LPD predominantly occurs in females of reproductive age; however, the pathogenesis of LPD is poorly understood. High levels of estrogen and progesterone, such as oral contraceptives, pregnancy, ovarian stimulation, estrogen-producing ovarian tumors, and uterine leiomyoma, have been described in most reported cases [5, 9, 12–15]. In this case with pregnancy, high levels of estrogen and progesterone stimulation also played an essential role in the development of LPD. Besides, the tumor cells were strongly positive for ER and PR in immunochemistry analysis, supporting the hypothesis that high levels of estrogen and progesterone playing an important role in the pathogenesis of LPD.

Immunohistochemical analysis of this case showed that SMA and Desmin were strongly positive, suggesting that LPD has similar molecular cytogenetic characteristics with uterine leiomyoma. However, LPD differentiates distinctly from uterine leiomyoma in phenotype. Uterine leiomyoma is obviously benign, whereas LPD has the quasi-malignant behavior. NGS might provide the potential molecular explanation that would explain this difference in phenotype. Compared with the common population, CNs of CDK4, MYC, NBN, and DAXX were all amplified for at least 4 times in this LPD. Immunochemistry of the four genes among uterine leiomyoma, LPD, and uterine leiomyosarcoma was implied. LPD and uterine leiomyosarcoma both were moderately and strongly positive for the four genes mentioned above, whereas uterine leiomyoma was slightly positive or negative. CN mutations might play an important role in the pathogenesis mechanism of LPD and identify LPD in phenotype from uterine leiomyoma. Further study is in urgent need to delineate the molecular mechanisms underlying the LPD phenotype. In addition, some literatures have confirmed that LPD will be followed by malignant transformation [16–18]. Based on the above results, we should pay attention to the potential malignancy of LPD during the treatment and follow-up of LPD.

Most importantly, we will discuss the feasibility and safety of pregnancy in patients with LPD. We searched PubMed database with key words of “leiomyomatosis peritonealis disseminata,” “peritoneal leiomyomatosis,” “leiomyomatosis,” “disseminated fibrosing deciduosis,” “LPD,” “pregnancy,” and “pregnant.” Sixteen cases of LPD during pregnancy with detailed clinical and follow-up information published from 1973 to 2012 were included for analysis [9, 13, 19–31], and the details were available in Table 3. The patient’s age was between 22 and 40 years old, and the history of pregnancy and childbirth seems to have no obvious correlation with the occurrence of LPD. There were three patients with a history of hysteromyoma resection, which may be one of the causes of LPD [20, 21, 29]. Ten cases of LPD patients without obvious clinical symptoms were delivered at full term [13, 19, 20, 23–27, 30, 31]; therefore, for the patients without obvious symptoms, close follow-up could be conducted without surgical treatment, but the patients should be fully informed of possible complications and malignant changes in the tumor. LPD was accidentally diagnosed in ten patients during the cesarean section due to obstetric reasons, such as fetal distress, abnormal labor process, and vulvar hematoma, and these complications were not directly related to LPD [13, 19, 20, 23, 25–28, 30, 31]. Abdominal pain is the most important complication of LPD during pregnancy, which may be related to the rapid growth and compression of the lesions [9, 22, 29]. The huge volume of LPD lesions could lead to abnormally increased pressure in the amniotic cavity, so PROM was relatively common [9, 19, 24], and in our case, the maximum diameter of the tumor reached 20 cm. LPD that occurred during pregnancy does not appear to have a significant adverse effect on newborns, except for complications related to preterm delivery [22, 24].

**Table 3:** The summary of LPD cases occurring during pregnancy

No.	Author	Age	History of hysteromy-omectomy	Obstetric history	Assisted reproductive technology	Gestational weeks	Complications	Operative methods	Fetal health	Follow-up (time)	Recurrence
1	Summa et al. [9]	29	No	G1P0	No	22^+6^	Abdominal emergency, fever, suspected preterm premature rupture of membranes	Explorative laparotomy and partial nodule resection(22^+6^ weeks) Cesarean section(28^+6^ weeks)	Sepsis, icterus and retinopathia II°	1 year	No
2	Dreyer et al. [13]	26	Unknown	G1P0	Unknown	Full term	Vulval haematoma	Explorative laparotomy	Healthy	Not applicable	Unknown
3	Hardman et al. [19]	33	Unknown	Unknown	Unknown	36	Premature rupture of membrane	Cesarean section and omental biopsies	Unknown	43 months	No
4	Hardman et al. [19]	36	Unknown	Unknown	Unknown	38^+5^	Placenta previa	Cesarean section and omental biopsies	Unknown	146 months	No
5	Aterman et al. [20]	22	Yes	Unknown	No	Full term	Fetal distress	Cesarean section and nodules biopsies	Unknown	4 months	No
6	Tanaka et al. [21]	40	Yes	Unknown	IVF-ET	Unknown	twin pregnancy	Cesarean section and nodules biopsies^1st^ Hysterectomy and nodules resection^2nd^	Unknown	8 months^1st^ 18 months^2nd^	Yes No
7	Valente et al. [22]	32	Unknown	G3P2	No	28	Abdominal pain, ascites	Explorative laparotomy, nodule resection, and cesarean section	Good condition at 9 months	9 months	No
8	Rubin et al. [23]	27	No	G1P0	No	Full term	Active phase arrest	Cesarean section and partial nodules resection	Unknown	6 months	Sarcoma diagnosed
9	Lim et al. [24]	22	Unknown	G4P1	Unknown	Full term^1st^ 35^2nd^	Premature rupture of membrane^2nd^	Cesarean section and nodules biopsies^1st and 2nd^	Unknown	20 months^1st^ 8 months^2nd^	Yes No
10	Pieslor et al. [25]	32	No	G2P1	No	Full term	No	Cesarean section	Healthy	Not applicable	Unknown
11	Nogales et al. [26]	34	Unknown	Unknown	Unknown	Full term	Prolonged labor	Cesarean section, total hysterectomy, and partial nodules resection	Unknown	Not applicable	Unknown
12	Parmley et al. [27]	36	Unknown	Unknown	Unknown	Full term	No	Elective tubal ligation and nodules resection	Unknown	2 years	No
13	Crosland DB [28]	29	Unknown	G2P1	Unknown	8	Severe hypertension	Suction curettage, omentectomy and nodules biopsies	NA	6 months	No
14	Deering et al. [29]	33	Yes (LPD)	G2P1	IVF-ET	10	Abdominal pain, hydronephrosis and hypertension	hysterectomy, bilateral salpingo-oophorectomy, radical pelvic lymph nodes dissection	NA	9 months	No
15	Kouakou et al. [30]	35	No	G4P1	No	Full term	Large fetus size	Cesarean section and omental biopsies	Healthy	2 months	No
16	Hoynck et al. [31]	35	No	Unknown	No	Full term	Fetal distress	Cesarean section, multiple biopsies, omentectomy, and right salpingectomy	Healthy	3 years	No
17	Our case	19	Yes	G1P0	No	32 + 3	Oligohydramnios and abdominal pain	Cesarean section and nodules resection	Healthy	25 months	No

In previous reports, LPD lesions could naturally shrink or disappear after delivery, and the tumor did not relapse during the reported follow-up period [19, 28, 31]; therefore, for patients without fertility requirements, radical surgery was unnecessary. However, there was limited literature on how patients with subsequent fertility requirements should be treated. The patient reported by Deering was diagnosed with LPD before pregnancy, and the lesion rapidly increased in a short period of time after receiving IVF-ET, suggesting that assisted reproductive technology may induce the occurrence and progress of LPD [29]. Lim OW reported a case of a pregnant patient with LPD who underwent only nodules biopsy at the first cesarean section, and the patient developed PROM at 35 weeks and recurrence of the LPD at the second pregnancy [24]. In our report, we suffered great difficulty and risk of complete removal of all visible lesions during the first cesarean delivery, and no lesions in the pelvic and abdominal cavity in the second cesarean section were found, suggesting that complete resection of the lesion may be beneficial for the subsequent pregnancy. However, more patients are needed to confirm this conclusion.

Finally, we will discuss the significant risk of LPD patients receiving assisted reproductive technology. In the case reported by Tanaka YO, the patient previously underwent laparoscopic myomectomy, followed by IVF-ET, and had a cesarean section due to twin pregnancy. LPD biopsy was performed during the cesarean section. However, the patient’s lesions continued to increase and finally received total hysterectomy and lesions resection 8 months after delivery, and no disease progression was found after 18 months of follow-up [21]. In the case reported by Deering, the patient had a history of LPD and confirmed the existence of the disease before receiving IVF-ET. After receiving IVF-ET, the lesions in the pelvic and abdominal cavity increased rapidly, and severe hydronephrosis occurred due to tumor compression. The pregnancy was terminated at 10 weeks of pregnancy because of the intolerance of the patient and more potential risks. The patient was treated with methotrexate and leuprolide, but the tumor did not shrink significantly; finally, the patient underwent a total hysterectomy and bilateral appendectomy, and radical resection of the lesions. In the subsequent follow-up, no recurrence of the disease was found [29]. The above two medical records reminded us that IVF-ET was a high-risk factor for LPD and could cause serious consequences. Assisted reproductive technology should be used with caution in this group of people.

In conclusion, LPD is an unusual intermediate between benign and malignant uterine smooth muscle tumors. We recommend that all visible lesions should be removed as completely as possible during surgery, which may be a very effective treatment plan in addition to radical surgery, and re-pregnancy may be feasible. Besides, assisted reproductive technology should be used with caution in LPD patients.

## Data Availability

All data generated or analyzed during this study are included in this published article.
